# Individual differences in processes of lifestyle changes among people with obesity: an acceptance and commitment therapy (ACT) intervention in a primary health care setting

**DOI:** 10.1017/S146342362000016X

**Published:** 2020-05-18

**Authors:** Kirsti Kasila, Suvi Vainio, Mari Punna, Päivi Lappalainen, Raimo Lappalainen, Kirsikka Kaipainen, Tarja Kettunen

**Affiliations:** 1 Faculty of Sport and Health Sciences, University of Jyvaskyla, Jyvaskyla, Finland; 2 Department of Psychology, University of Jyvaskyla, Jyvaskyla, Finland; 3 Headsted Ltd, Tampere, Finland; 4 Central Finland Health Care District, Jyväskylä, Finland

**Keywords:** acceptance and commitment therapy, learning, lifestyle change, obesity, self-regulation, web-based intervention

## Abstract

**Aim:**

To explore what thoughts, feelings, and learning processes were involved in obese participants’ lifestyle change during an acceptance and commitment therapy (ACT) lifestyle intervention delivered in primary health care.

**Background:**

Previous studies have revealed that lifestyle interventions are effective at promoting initial weight loss, but reduced weight is often difficult to sustain because of the failure to maintain healthy lifestyle changes. Achieving and maintaining lifestyle changes requires to learn self-regulation skills. ACT-based lifestyle interventions combine many self-regulatory skill factors, and the results from previous studies are promising. Research on the individual learning processes of lifestyle change is still needed.

**Methods:**

This study investigated a subset of data from a larger web-based lifestyle intervention. This subset consisted of online logbooks written by 17 obese participants (*n* = 17, body mass index mean 41.26 kg/m^2^) during the six-week online module. The logbooks were analyzed via data-driven content analysis.

**Findings:**

Four groups were identified based on the participants being at different phases in their lifestyle changes: stuck with barriers, slowly forward, reflective and hardworking, and convincingly forward with the help of concrete goals. Differences between the groups were manifested in personal barriers, goal setting, training of mindfulness and acceptance, and achieving healthy actions. The ACT-based lifestyle intervention offered participants an opportunity to reflect on how their thoughts and feelings may hinder healthy lifestyle changes and provided tools for learning psychological flexibility.

## Introduction

Obesity is a universal risk factor for many noncommunicable diseases, such as cardiovascular diseases and diabetes (WHO, 2011). Previous studies have revealed that behavioral interventions are effective at promoting initial weight loss (Wu *et al*., 2009; Olander *et al*., 2013), but reduced weight is often difficult to sustain because of the failure to maintain healthy lifestyle changes (Dombrowski *et al*., 2014). The barriers toward lifestyle change that obese people struggle with obviously complicate the change process. The key barriers toward lifestyle changes are poor motivation, lack of time, environmental, societal and social pressures, health and psychical limitations, negative thoughts/moods, socioeconomic constraints, gaps in knowledge, and the lack of enjoyment of exercise (Burgess *et al*., 2017a). Therefore, in addition to essential components like diet and physical activity, interventions should take individual barriers into consideration and address them appropriately (Burgess *et al*., 2017a).

On the other hand, previous studies have found that many self-factors, such as positive body image, self-efficacy, and autonomous motivation, and self-regulatory skills and techniques, such as goal setting, action planning, and self-monitoring, are associated with successful lifestyle changes, weight outcomes, and weight management (Greaves *et al*., 2011; 2017; Gupta *et al*., 2014; Teixeira *et al*., 2015; Burgess *et al*., 2017b). Over the past few years, acceptance and commitment therapy (ACT), a third-generation cognitive behavioral therapy, has been applied within the context of lifestyle intervention by combining many self-factors and self-regulatory skills and processes (Hooper and Larsson, 2015; Hayes, 2016; Rogers *et al*., 2017). At its core, ACT is designed to increase psychological flexibility (Hayes, 2016), which is defined as the ability to identify and deal with barriers to change, and as the ability to take responsibility for one’s well-being (Hayes *et al*., 2006).

Psychological flexibility is established through six core learning processes. These processes can be divided into two types of processes: commitment and behavior change processes called values and committed actions and mindfulness and acceptance processes called self as context, present moment, acceptance, and defusion. They overlap with each other and act together to enhance psychological flexibility. The values clarification process aims to provide the person with her/his most important directions in life, including effective goal setting, commitment to taking these small steps, and dealing with setbacks (Hayes *et al*., 2006; 2013). Committed action refers to helping the person to actively pursue actions linked to chosen personal values. While engaging in this process, the person will inevitably encounter difficult thoughts and feelings. The aim of the mindfulness and acceptance processes is therefore to help the person to become aware and cope with both internal (e.g., thoughts and feelings) and external barriers (e.g., time, money and opportunity) (Flaxman *et al*., 2011). Self as context is about adopting a sense of self where a person’s self is not defined as the thoughts, feelings, and sensations that she/he is experiencing, but rather as the person who is having or noticing various thoughts, feelings, and sensations (Flaxman *et al*., 2011). The aim is not to remove the conceptual thinking but to change the impact of thoughts and feelings on one’s sense of self (Hayes *et al*., 2012). Contact with the present moment is about fostering an accepting and non-judging awareness of one’s thoughts, feelings, and sensations as they unfold from one moment to the next. Attending more to the present moment, noticing how effectively or ineffectively one is behaving in the here and now, a person is better able to change her/his behavior (Flaxman *et al*., 2011). Acceptance involves willingness to accept one’s private experiences (thoughts, feelings, and sensations) – even the difficult ones – without trying to change, avoid, or suppress them (Hayes et al., 2006; Hayes, 2016). Defusion is about learning to step back and watch one’s thinking without getting overwhelmed by them. It is a process that reduces the impact of thoughts and feelings on a person’s behavior (Twohig, 2012). The aim of this process is not to reduce or suppress the thoughts and feelings but to reduce their credibility and impact on behavior (Hayes *et al*., 2006).

Training acceptance-based self-regulation skills has been found to be associated with positive short-term and long-term weight outcomes (Rogers *et al*., 2017). Yet the need for research to address the processes of change in ACT-based lifestyle interventions remains (Hooper and Larsson, 2015; Rogers *et al*., 2017). Research on the individual learning processes of lifestyle change is needed. This qualitative study focused on a participant point of view to illustrate the lifestyle change during an ACT-based intervention. In particular, the aim was to explore what thoughts, feelings, and learning processes were involved in obese participants’ lifestyle change during an ACT-based online intervention delivered in primary health care.

## Method

This study was a part of a larger 24-month lifestyle intervention for overweight and obese people in Central Finland. The web-based lifestyle intervention involved 10 lay health workers (LHWs) who served as layman behavioral experts. They had completed an eight-month training program in the Central Finland Health Care District, and their expertise was focused on a large variety of chronic diseases and health problems. Furthermore, they had been trained as online tutors and supporters for this study. Prior to the intervention, the LHWs pre-tested all of the exercises and tasks of the intervention. Over the course of 24 months, the intervention contained three six-week online modules, five group meetings, and four phone calls. The study reported in this paper focused on the first six-week online module.

In the ACT-based online modules, participants worked with a different theme every week. Table 1 describes the contents of the first six-week online module. After each week, there was a weekly task in which participants wrote down their reflections and experiences about the content and exercises of the particular week. The participants sent their weekly tasks to their LHW tutor, who provided them with encouraging feedback. The reflections and considerations regarding weekly tasks formed the content of the participants’ logbooks. The group meetings consisted of group discussions and were planned and carried out in cooperation with health care professionals and LHWs. The LHWs’ phone calls were used to improve participants’ adherence to intervention.

**Table 1.** The six-week online module

Week	Theme	Titles of the exercises	Week tasks
1	Identify your values. What is important in your life?	Curtains in the window The most important in life The 80th birthday Values	What kind of life do you want to live and what is important to you? Are you acting according to your values?
2	Act. Take small steps to improve your well-being.	Do it now Passengers on a bus Obstacle race	What steps could you take today to increase your well-being? Observe what kinds of barriers occur when you’re trying to make changes.
3	Increase your awareness. Do things mindfully.	Observe five things Follow your breath Mindful eating Mindful listening Mindful walking Everyday chores Leaves in a stream	Do something mindfully that you usually do automatically. Use all of your senses. What steps have you taken toward well-being?
4	Observe and monitor yourself. Learn to know yourself.	Sky and weather Deep down, who are you? House and furniture Terrier thoughts Observer	In what situations are you stuck in your own stories and thoughts? Distance yourself from your thoughts. Do you realize which thoughts prevent or promote your change? What value-based actions have you done this week?
5	Watch your thinking.	Hands in front of eyes Little man Observe Doughnuts Make room for your thoughts and feelings	What are the feelings and thoughts about yourself that you want to take distance from? Try to identify them whenever they occur. What value-based actions have you done this week?
6	Let go and accept things you can’t change.	Tug of war with a monster Broken machine Rock on the beach Observer Compassion toward yourself	Is there something in your life that you should try to let go and accept? Practice acceptance toward one thing. What value-based actions have you done this week?

## Participants

The participants were recruited to the lifestyle intervention via 12 health care centers in Central Finland. During their clinical appointments, the health care professionals (nurses and physicians) recruited voluntary adult patients who met the inclusion criteria [body mass index (BMI) ≥ 25 kg/m^2^] and were identified as potentially benefitting from support to make health-related lifestyle changes to participate in the study. Of the 177 participants who completed the whole intervention, 84% (*n* = 148) were female and 87% (*n* = 154) were obese (BMI ≥ 30 kg/m^2^). The participants of the intervention formed 16 groups (eight to fifteen individuals/group) regionally in Central Finland, and the groups started at different times in each region. Each LHW was responsible for tutoring one or two groups. The participants’ consent to participate in the study was requested personally from each individual. Ethical approval for the study was received from the Central Finland Health Care District Ethics Committee.

### Data collection and analysis

This qualitative study focused on the first six-week online module of the lifestyle intervention and the first two groups who started the intervention (*n* = 17; 16 females; BMI mean 41.26 kg/m^2^; mean age 45.47 years). This focus was chosen to allow in-depth investigation of learning processes in the initial phase of lifestyle change. The logbooks of these 17 obese participants were collected from the database of the web-based intervention, and altogether these contained about 9000 words. The logbook data were analyzed through qualitative content analysis (Schreier, 2012). First, the analyzing frame from every participant’s logbook was constructed by paraphrasing all relevant units, condensing them into a summarized expression and finally creating subcategories based on similarities in condensed expressions. Second, the 17 constructed analyzing frames were compared and six core main categories were identified (current situation, goal setting, promoters, barriers, training of mindfulness and acceptance, and self-reported healthy actions) that combined all of the data. Based on the identified differences in these main categories among the participants, four groups were defined (Table 2).

**Table 2.** Characteristics of the constructed groups

Group/main categories	Current situation	Promoters	Barriers	Goal setting	Training of mindfulness and acceptance	Self-reported healthy actions
G1: Stuck with barriers (*n* = 3)	Partly clarified values Realization that their own well-being has been neglected Life is good right now	Pleasant activities	Worries about coping Cannot do things by themselves Inefficiency Chocolate Their own obesity Other people’s comments about weight	Promises to exercise Self-development in small steps Recovery from surgery	Realizing that one is not always present in everyday life No statements about implementing exercises in everyday life	-
G2: Slowly forward (*n* = 4)	Values stated clearly Clarification of wishes Realizing that weight loss requires actions	-	Fear, feelings of insecurity, and difficulty of change Negative feelings about themselves Exhaustion and fatigue Being stuck Something else in life takes away resources	Changes in the future (afterwards) Investment in the everyday things (e.g., washing dishes daily) Small goals toward better life	Practicing mindfulness – the skill of being present Realizing that you can get a new perspective on your thoughts and feelings Exercises evoked negative emotions For some, acceptance is hard; for others, feelings of achievable acceptance	Paying attention to oneself More exercise Better eating with small changes
G3: Reflective and hardworking (*n* = 7)	Values stated clearly Clarification of wishes Realizing that own actions contradict own values Need for more exercise and better eating Current situation is okay	Pleasant activities Support from others Meaningful job Positive attitude	Negative feelings about themselves Difficulty to start Struggling with food Other people and situations in life take away resources	Possibly more exercising Possibly better eating Concrete goals to exercise more Concrete goals to eat better Weight control Improving oneself	Practicing mindfulness – being present in everyday life By doing exercises – a more positive way of thinking Awareness about themselves and their own actions increased Self-acceptance Acceptance is hard for some For some, no need for acceptance	Better eating and drinking More exercise Weight loss Improving as a person More time for family
G4: Convincingly forward with the help of concrete goals (*n* = 3)	Wishes and values clearly stated	-	Sporadic barriers: Disbelief in oneself Getting started is difficult Something else in life takes away resources	Concrete goals to increase exercise Concrete goals to eat better Concrete goals to have more time for family Concrete goals to improve oneself	Realizing that contact with the present moment is sometimes difficult Practicing contact with present moment in everyday life Exercises have helped to clear thoughts Realizing that attitude matters Self-acceptance	More exercise Succeeding and exceeding oneself

## Results

The four constructed groups that described the participants’ individual progress of lifestyle change processes were named *stuck with barriers*, *slowly forward*, *reflective and hardworking*, and *convincingly forward through concrete goals* (Table 2).

### Group 1: Stuck with barriers

In this group, personal values and wishes were not clearly stated. However, the participants admitted that they had not taken care of their own well-being, indicating that they were aware of their behavior. They reported that their overall life situation was basically still okay at that moment. This group found strength and positivity in their lives in pleasant activities and hobbies. However, there were more barriers than promoters, and the participants in this group reported more barriers than participants in other groups. The participants were concerned about their own coping skills; they felt that they were not able to achieve the change alone without external support and many felt inefficient. Emotional eating was seen as a problem, for example, chocolate was one way to ease unpleasant feelings. Obesity and the difficulty to lose weight was seen as a big barrier. Comments from other people about weight also caused negative feelings. All in all, the barriers appeared in many reflections:

Honestly, I haven’t done anything concrete. Honestly, I don’t know where to start. (Participant 3)

My thoughts are now so full, I stress about school, unfinished business, and about how I’m dragging with this journey. (Participant 2)

… I feel bad and chocolate makes me feel better. (Participant 1)

Only a few goals were set in this group, and they were mostly promises about doing things later: “But, I promise, that next week, I will go for a little jog every day…” (Participant 1). This group had thoughts about being active and doing physical exercises, and they realized that they were not always present or capable of accepting themselves. However, there were no statements about implementing the mindfulness and acceptance exercises in everyday life, nor of getting a new perspective on their thinking. During the six-week online module, there were no self-reported healthy actions or successes in this group.

### Group 2: Slowly forward

This group was defined by somewhat slow movement toward change. In this group, value and wish clarification were clear. Losing weight was separately mentioned as an important thing in life and also as a wish for the future. Participants in this group mainly wished for a more enjoyable life than they were living at the present moment. Some in this group wished for a life without fear, uncertainty, or pain:

I would like to be able to do what others can, but it is not possible because I’m overweight. (Participant 4)

The participants in this group wanted to live a healthy life and be in charge. They wanted to improve themselves, be more energetic, and find the strength to succeed in change. Participants realized that they needed to start making changes in their lives. However, the participants in this group found barriers in the environment and in other people. For example, the fear of what other people might think about their obesity was present. Feelings of fear concerning work or family situations also emerged. Their own resources had gone into taking care of other people. The image of the self was mainly poor, and there were feelings of uncertainty and difficulty in getting started. In addition, fatigue and exhaustion were seen as big challenges. Halting and slower progress stemmed from feelings of being stuck and not moving forward: “I haven’t lost much weight now…now, I’m stuck, and it doesn’t really feel that anything is happening…” (Participant 7).

The participants in this group set many different goals, some of which were set in the near future and some later. Goals regarding diet and exercise were set further away. Goals regarding self-improvement, for example, concentrating more on oneself, were more present. Starting with minor lifestyle changes and concentrating, for example, on everyday chores such as washing dishes, were also present in the goal setting.

I will try to think more about myself and start to take care of myself… (Participant 6)

I will walk to the mailbox every day. (Participant 4)

While doing the mindfulness exercises, this group realized that they can get a new perspective on thinking and acting: “Interesting what awareness can do…things can actually be seen in a different way” (Participant 4). They also implemented mindfulness and acceptance exercises in their everyday life. For this group, the exercises awakened some negative emotions. For instance, while doing the exercises, painful memories from the past were activated. Regarding acceptance, there were careful improvements, but also many challenges: “…maybe little by little I will start to consider and analyze my thoughts better…” (Participant 5)

This group paid more attention to their own lifestyle actions. Slower movement toward lifestyle change was shown in the healthy actions, where many successes were little choices toward the better: “I really didn’t care or have the strength to go out today, but I still went for a half-hour jog…” (Participant 6).

### Group 3: Reflective and hardworking

The participants in this group conscientiously completed the exercises of the online module with reflection and consideration. This group had a clear definition of desire and values. They wished for a healthy, happy, calm, and balanced life. They also wished to improve as a person, but in addition, they wished to be able to take care of their family. The participants in this group valued health, family, friends, and leisure time. In addition, this group valued work. For some participants in this group, the current situation was seen as acceptable, but at the same time, participants stated that they realized that they were not acting according to their values. They reflected on what they should do differently and stated that they should exercise more, eat better, and develop some self-control.

The participants in this group considered many promoters for their change. Pleasant activities, such as hobbies and work, were seen to be enjoyable and meaningful. Getting support from family and friends was also seen as a promoter for change. In this group, participants also had a positive attitude toward the process of lifestyle change: “…I have come to a conclusion that hey, I have managed to quit smoking, so why couldn’t I accomplish something else?” (Participant 14)

Along with many promoters, a number of barriers were also identified. There were some implications of negative feelings and thoughts about the self. Participants had feelings of failure and inferiority. Struggling with food was also seen as a barrier; there were statements that it was hard not to eat sweets or restrain from eating extra servings. Regardless of the barriers, this group sets many goals toward change, many of which were set with confidence:

Tomorrow, I’m going to the water aerobics class. (Participant 9)

I’m going to drink more water; I’m taking a bottle of water to work! (Participant 11)

However, some goals were set more doubtfully, or they were not so straightforward: “If maybe today I go for a little jog.” (Participant 10). With the help of the mindfulness exercises, this group tried to be more present in their everyday lives. The exercises helped them to be more aware of themselves and their actions. Participants reported that their thinking had started to shift in a more positive direction: “I have become aware [of the fact] that good things can happen to me too.” (Participant 12)

In this group, the need or importance of the acceptance exercises was not clear. Acceptance was hard or was not seen as important. On the other hand, there were also signs of improvements in acceptance if it was seen as important: “With him (friend that passed away), I never felt inferior, fat, or stupid. I was enough as I am. So maybe I should accept myself too.” (Participant 8). This group also achieved a lot of healthy actions. The healthy actions included exercising more, eating better, losing weight, concentrating more on oneself, and finding more time for one’s family.

### Group 4: Convincingly forward with the help of concrete goals

The participants in this group were well aware of their personal values and had a clear commitment for lifestyle changes. They wished for a healthy, happy, balanced, and regular life. They also wanted to improve themselves as people. Health was seen as an important issue. Family, relationship, and friends were significant, yet time for themselves was also valued. Some barriers were identified. Some participants found it hard to believe in themselves, and it was difficult to get started. There were also some aspects of life that might be obstacles on their journey. For example, the Christmas rush was seen as a barrier. Regardless of the barriers, this group sets clear goals toward the change. They stated their goals convincingly and clearly. They wanted to exercise more, eat better, make time for their family, and improve as a person.

The mindfulness and acceptance exercises helped the participants to realize that they were not always present in their lives. Participants implemented the exercises in their everyday lives and practiced the skill of staying present in the moment. These exercises made the participants realize how much the skill of being more in the here and now can affect their life, and how much attitude actually matters. Participants felt that the exercises helped them to see more clearly and self-acceptance was also achieved:

Exercises have already helped a lot. As if I could now see the path I should take, when earlier, I circled around. (Participant 15)

I accept the fact that my body isn’t the same [as] when I was 20. (Participant 17)

The participants in this group made clear healthy actions toward change. For example, they reported that they had exercised more. They also managed to exceed themselves in many ways and were happy about their journey.

For this week’s healthy action, I went swimming three times. It was very nice. I have avoided swimming, even though I like it, because I’m not really okay with my tummy in a swimsuit. But now, I silenced the ‘little man’ (in my head), and it was worth it. (Participant 15*)*

## Discussion

This qualitative study brings novel insight to the learning processes and progress of obese people’s lifestyle change that is multifaceted and influenced by many factors. All the participants reported signs of starting the change process. Theoretically, all participants were in either the intention or action phase in their change process (Schwarzer *et al*., 2011). However, variation in levels of personal barriers, goal setting, practicing exercises, self-acceptance, and health actions were observed.

The participants in two groups (G3, G4) were most clearly in the action phase as they reported concrete goals, a high degree of practicing mindfulness and presence in everyday life, improvements in self-acceptance, and health actions. The participants in other groups (G1, G2) had a willingness to change their lifestyle, and they reported goal setting although some goals remained just wishes. Furthermore, the difficulty of barriers occupied significant space in their thinking, and they practiced less self-regulation skills than the other groups did. However, among those who practiced mindfulness and other experiential exercises, slower movement toward change was noticeable. The participants reported poor self-image and feelings of uncertainty, but on the other hand, they learned that they can get another perspective on their way of thinking. It was observable that these participants attempted to transform their intention into action during the first online module of the intervention.

One critical point in the change process seems to be practicing and learning coping skills for overcoming the barriers (Forman and Butryn, 2015; Texeira *et al*., 2015; Greaves *et al*., 2017). Reflection on barriers has been found to be crucial in obese peoples’ adherence and maintenance of healthy lifestyle changes (Dombrowski *et al*., 2014; Burgess *et al*., 2017a). The exercises of the web intervention enabled the participants to reflect on both the barriers and promoters of behavior change. Most barriers concerned negative thoughts and feelings associated with one’s own obesity, appearance, and feelings of insecurity. It is known that individuals’ negative thoughts and emotions about themselves may have a strong impact on self-efficacy and behavior (Twohig, 2012; Gupta *et al*. 2014; Burgess *et al*., 2017a), and the key point in the acceptance and change process is to understand one’s own conceptual thinking (Hayes *et al*., 2006; 2012). By practicing the exercises they were provided via the web-based ACT program, they became more aware and realized that it is possible to learn a new perspective on one’s thinking and to accept oneself. For some, the exercises also evoked negative feelings about, for example, their own obesity, possibly indicating the difficulty to tolerate thoughts and feelings about their obesity and body image (Teixeira *et al*., 2015; Greaves *et al*., 2017). This suggests that they may not yet be willing to accept the distressing thoughts that will inevitably emerge regarding their obesity and body image. Therefore, more training in acceptance and other processes may be needed.

Choosing a valued direction for action (Hayes *et al*., 2012) was also a differentiating factor for the phases of lifestyle change. The exercises of the intervention encouraged the participants to clarify their values and to take actions toward them. Value work was mainly considered easy, but difficulties were observable in prioritizing personal values. In some cases, value clarification helped the participants to realize that their current actions were against their own values. Goal setting has been shown to predict successful healthy behavior and weight maintenance (Greaves *et al*., 2011; Olander *et al*., 2013; Teixeira *et al*., 2015). It is known that the capability to identify personal values and the effort to act toward achieving them precedes committed action (Hayes *et al*., 2013).

Despite the fact that the first two groups of the first six-week online module formed the data, the analysis was able to identify experiences important to acknowledge in obese participants’ lifestyle change learning. These data were selected for this study to allow in-depth analysis of learning processes and because the results were immediately utilized in the creation of the intervention’s subsequent online modules. When the LHWs tested the exercises of the online module, they reported that some of the mindfulness and acceptance exercises were difficult. Therefore, it was important to obtain information about the feasibility of the online exercises for obese participants at the beginning of the study. The content analysis produced both individual and group-level information regarding the research aim. The condensed analysis frame, including six core main categories, was exhaustive, and all the categories created were internally consistent. To ensure that the categories reflected the data, the analysis constantly moved back and forth between the original data and the categories created. Using extracts from the logbook accounts of the participants increases the dependability of the results by grounding interpretations within the data. The reader can therefore assess the trustworthiness of the analysis (Schreier, 2012).

### Conclusion and practice implications

In this study, the lifestyle intervention was implemented as a part of normal work in primary health care, so it is directly applicable to practice. The findings revealed four distinct groups in different phases of lifestyle changes, and three main advantages of the ACT-based intervention: (1) it provides tools for dealing with problematic thoughts and feelings that are often a barrier to lifestyle changes; (2) it provides assistance in identifying personal values and in setting concrete goals when planning lifestyle changes; and (3) it encourages taking value-based actions. Hence, the different types of exercises in the intervention can be suitable for learning a variety of self-regulation skills.

The study data were collected from the first six-week module of the whole intervention. Therefore, the studied time period may be too short for making sustainable lifestyle changes. In the future, a larger intervention study aims to examine the learning of self-regulation skills over a longer period to observe the development of these skills and their association with health behavior and weight outcomes. The results of this study could serve as a framework for a future analysis that considers all three online modules of the whole intervention.
